# Photo-Switchable Aggregation-Induced Emission of Bisthienylethene-Dipyrimido[2,1-*b*][1,3]benzothiazole Triad

**DOI:** 10.3390/molecules26175382

**Published:** 2021-09-04

**Authors:** Shan-Shan Gong, Chun-Hong Zheng, Zhen-Zhen Chen, Dong-Zhao Yang, Mei Chi, Shou-Zhi Pu, Qi Sun

**Affiliations:** 1 College of Chemistry, Nanchang University, Nanchang 330031, China; gongshanshan@jxstnu.edu.cn; 2 Jiangxi Key Laboratory of Organic Chemistry, Jiangxi Science and Technology Normal University, Nanchang 330013, China; ezirobot@163.com (C.-H.Z.); chenzhenzhen5@126.com (Z.-Z.C.); yangdongzhao1@126.com (D.-Z.Y.); chimei5@126.com (M.C.); 3 Department of Ecology and Environment, Yuzhang Normal University, Nanchang 330103, China

**Keywords:** AIE, photochromism, pyrimido[2,1-*b*][1,3]benzothiazole, diarylethene, photoswitch

## Abstract

A bisthienylethene-dipyrimido[2,1-*b*][1,3]benzothiazole (**BTE-2PBT**) triad has been designed and synthesized based on our recent discovery of PBTs as atypical propeller-shaped novel AIEgens. The triad not only maintains the photochromic properties of BTE moiety in solution, film, and solid state but also exhibits remarkable AIE properties. Moreover, the fluorescence of **BTE-2PBT** PMMA film could be modulated with high contrast by alternate UV and visible light irradiation. Photoerasing, rewriting, and non-destructive readout of fluorescent images on **BTE-2PBT** PMMA film well demonstrate its potential application as optical memory media.

## 1. Introduction

Photochromic diarylethenes have been acknowledged for their excellent photochemical reactivity, thermal stability, and fatigue resistance [1,2] and widely applied as photo-responsive materials in a variety of photonic devices [3]. Generally, the open-ring isomers of diarylethenes are weakly fluorescent depending on the nature of attached heteroaryl rings, but the fluorescence is typically quenched due to the formation of a larger conjugation system upon photo-induced ring closure. Therefore, diarylethene alone is not an ideal photo-switchable fluorophore. However, the huge absorption shift from 250–330 nm to 500–650 nm upon cyclization enables diarylethenes to function as photo-responsive energy acceptors in fluorescence resonance energy transfer (FRET) pairs. The photo-addressable fluorescence observed in diarylethene-fluorophore dyads [4,5] confirmed that the fluorescence modulation largely depends on the photoconversion ratio (PR) of diarylethene moiety. These photo-switchable fluorophores have been applied to labelling of biomolecules [6] and nanoparticles [7] to enable fluorescence switching with UV and visible light. However, most of these fluorescent photoswitches inevitably suffer from aggregation-caused quenching (ACQ), which greatly limits their applications as high-density optical materials [8].

The discovery of aggregation-induced emission (AIE) [9,10,11] has brought a new window to overcome the ACQ problem. It has been reported that the bisthienylethene-diquinolinemalononitrile triad [12] and bisthienylethene-dicoumarin triad [13] exhibited AIE properties. However, their photo-controlled modulation of solid-state fluorescence was not well realized. Currently, only a limited number of dyads and triads, which directly connect a bisthienylethene (BTE) with one or two AIEgens, such as *trans*-cyanostilbene [14,15], tetraphenylethene [16,17,18], and bispyridine salt [19], have been obtained as photo-switchable AIE materials [20,21].

Recently, our research group discovered pyrimido[2,1-*b*][1,3]benzothiazoles (PBTs) as atypical propeller-shaped novel AIEgens with full-color tunability, excellent solid-state fluorescence quantum yields, and high crystallizability [22]. Herein, we report the design and synthesis of a novel bisthienylethene-dipyrimido[2,1-*b*][1,3]benzothiazole (**BTE-2PBT**) triad. The novel triad not only well maintains its photochromic properties in solution, film, and solid state but also exhibits remarkable AIE properties. More importantly, the fluorescence of **BTE-2PBT** triad could be reversibly modulated with high contrast in solid state by UV and visible light irradiation, making **BTE-2PBT** triad a promising candidate for the application as photo-erasible optical memory media.

## 2. Results and Discussion

### 2.1. Synthesis of **BTE-2PBT** Triad

**Scheme 1:**
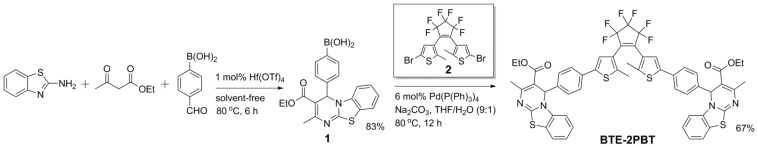
Synthetic route for **BTE-2PBT** triad.

As shown in Scheme 1, boronic acid-modified PBT compound (**1**) was synthesized via the Hf(OTf)_4_-catalyzed three-component reaction (3CR) of 4-formylphenylboronic acid, 2-aminobenzothiazole, and ethyl acetoacetate under solvent-free conditions [23]. The presence of boronic acid was well tolerated by the Hf(OTf)_4_-catalyzed 3CR, and **1** was obtained in 83% yield over 6 h. The Suzuki coupling of 2.5 equiv of **1** with 1 equiv of dibromobisthienylethene (**2**) in THF/H_2_O mixture (9:1, *v*/*v*) at 80 °C for 12 h afforded the desired **BTE-2PBT** triad in 67% yield as a light greenish powder. The NMR and HRMS spectra are provided in the Supplementary Materials. 

### 2.2. Photochromism of **BTE-2PBT** in Solution, Film, and Solid State

**Figure 1:**
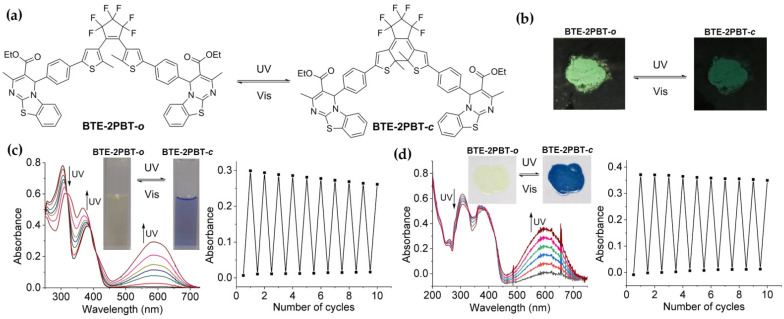
Photochromic properties of **BTE-2PBT** triad. (**a**) Photochromic reactions of **BTE-2PBT**; (**b**) Photochromism of **BTE-2PBT** in solid state; (**c**) Absorption change and fatigue resistance of **BTE-2PBT** in hexane (2.0 × 10^−5^ M) upon photochromism; (**d**) Absorption change and fatigue resistance of **BTE-2PBT** in PMMA film (5 wt %) upon photochromism.

The absorption maxima of the open-ring form of the triad (**BTE-2PBT-*o***) were observed at 305 nm and 380 nm corresponding to the BTE and PBT moieties, respectively. As shown in Figure 1a,c, irradiation of **BTE-2PBT-*o*** with UV light (254 nm) resulted in emergence and augmentation of a new absorption band at 584 nm with a color change from light yellow to blue due to the formation of closed-ring isomer (**BTE-2PBT-*c***). The blue color faded to light yellow upon irradiation with visible light (>510 nm), and the absorption spectrum returned to the initial state of **BTE-2PBT-*o***. The reversible color change along with isosbestic points at 319 nm, 392 nm, and 409 nm well demonstrated the bistable photochromism of the **BTE-2PBT** triad.

The key photochromic parameters including cyclization (*Φ*_o-c_) and cycloreversion (*Φ*_c-o_) quantum yields and photoconversion ratios (PRs) of **BTE-2PBT** triad in the photostationary state (PSS) in hexane (Table 1) were determined according to a known method [24,25]. Compared to unmodified perfluorobisthienylethene (**3**), **BTE-2PBT** exhibits bathochromically shifted λ_ab,o_ and λ_ab,c_ due to the extension of π-conjugation. Meanwhile, attachment of two PBT moieties also results in loweredcyclization and cycloreversion quantum yields. The PR of **BTE-2PBT** in PSS determined by analytical HPLC is 73%. In addition, **BTE-2PBT** could be reversibly switched between the two isomeric states without significant degradation for at least 10 times upon alternate irradiation with UV and visible light.

The **BTE-2PBT** triad in PMMA film (Figure 1d) also exhibits similar photochromic properties. The light yellow PMMA film of **BTE-2PBT-*o*** turned into blue upon irradiation with UV. The blue **BTE-2PBT-*c*** film could be completely bleached to the original color when exposed to visible light (>510 nm). The photoreaction parameters, such as absorption maxima of **BTE-2PBT-*o*** (306 nm and 385 nm) and **BTE-2PBT-*c*** (602 nm) and isosbestic points (327 nm and 421 nm), all slightly redshift in PMMA. As expected, the PMMA film of **BTE-2PBT** showed better fatigue resistance than its solution by insulating the photochromic compound from external oxidizing agents. Moreover, amorphous **BTE-2PBT** powder could also be reversibly switched between light green and dark green via photo-induced cyclization and cycloreversion (Figure 1b). These results indicate that the photochromic properties of BTE could be maintained when two PBT appendages are installed.

### 2.3. Photophysical and AIE Properties of **BTE-2PBT**

The absorption and photoluminescence (PL) properties of **BTE-2PBT** in solution, solid state, and film are summarized in Table 2. The absorption peak of **BTE-2PBT** in hexane appearsat 388 nm and arises from π–π* transition. The absorption peak of film sample shows up at 385 nm, whereas that of solid sample redshifts to 413 nm.

The data in Table 2 show that the triad is very dim in solution, but exhibits significant fluorescence in solid state and film when excited by 365 nm UV. The fluorescence lifetimes (τ) of **BTE-2PBT** in solid state and film show correlation with their fluorescence quantum yields. Comparison with a reference PBT AIEgen (**4**) shows that the excitation and emission wavelengths of PBT moieties in the triad are not affected by appending to BTE. However, the fluorescence quantum yields drop notably, possibly due to the enlarged molecular size, which may leave more space for intramolecular motion of the small ethyl ester rotor [22].

**Figure 2:**
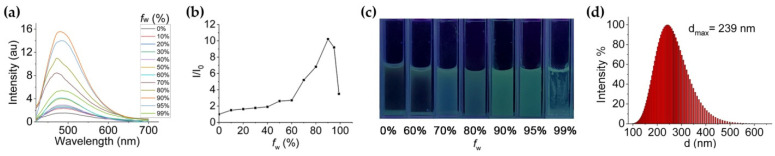
AIE properties of **BTE-2PBT** triad. (**a**) PL spectra of **BTE-2PBT** in THF and water/THF mixtures with different water fractions (λ_ex_ = 365 nm); (**b**) Plot of *I*/*I_0_* value vs. water fraction; (**c**) Photographs of **BTE-2PBT** in different water fractions taken under 365 nm UV; (**d**) Dynamic light scattering (DLS) of **BTE-2PBT** in 90% water/THF mixture.

The more detailed aggregation-induced emission properties of **BTE-2PBT** was investigated by gradually increasing the water fraction in its THF solution. As shown in Figure 2, when water content was elevated to 70%, the solution of the triad began to exhibit strong green fluorescence due to the formation of nanoaggregates. The emission reached its maximum (11 fold) when *f*_w_ was increased to 90%. Dynamic light scattering (DLS) method determined that the particle size upon aggregation in 90% water/THF mixture distributed between 100 nm and 500 nm with a maximum intensity at 239 nm. Further increase of water content led to declined fluorescence emission due to precipitation of **BTE-2PBT**. It is noteworthy that the maximum λ_em_ in 99% water only redshifts 10 nm compared to that in absolute THF. The insensitivity to solvent polarities indicates that locally excited (LE) state dominates the excitation of **BTE-2PBT** triad [22].

### 2.4. Photo-Switchable AIE Properties and Application of **BTE-2PBT**

**Figure 3:**
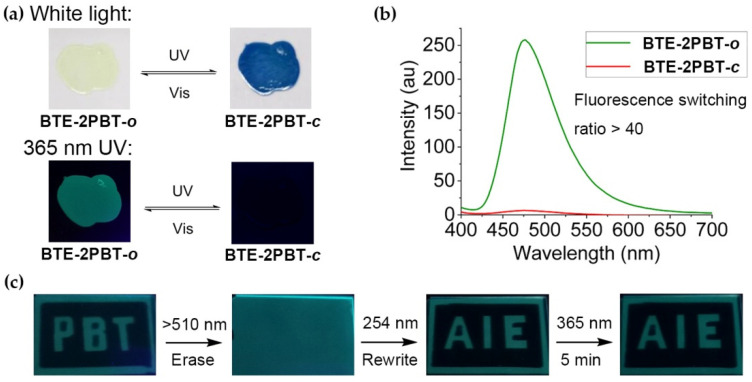
Photo-switchable AIE properties and application of **BTE-2PBT** triad. (**a**) Photographs of **BTE-2PBT** PMMA film illuminated with alternate 254 nm UV and >510 nm visible light taken under white light or 365 nm UV; (**b**) PL spectra of **BTE-2PBT** PMMA film at PSS illuminated with alternate 254 nm UV and >510 nm visible light (λ_ex_ = 365 nm); (**c**) Photoerasing, rewriting, and non-destructive reading of fluorescent images on **BTE-2PBT** PMMA film.

As **BTE-2PBT** triad maintains the reversible photochromic and AIE properties of the parent BTE and PBT moieties, we explored the possibility to modulate its solid-state fluorescence with photo-induced isomerizations. When the PMMA film containing 5 wt% **BTE-2PBT** was irradiated with 254 nm UV, its light yellow color turned into blue due to photochromism. Meanwhile, the green fluorescence of **BTE-2PBT-*o*** was almost completely quenched upon photocyclization (Figure 3a). The measurement of fluorescence intensity of **BTE-2PBT** PMMA film at “ON” and “OFF” states showed that 97.6% of open-ring state fluorescence was quenched upon photo-induced ring closure. The calculated fluorescence switching ratio is >40 (Figure 3b). The ultra high contrast could be ascribed to the combined quenching effects of both intra- and intermolecular energy transfer in the solid phase.

The application of **BTE-2PBT** PMMA film as erasible optical memory media was conducted by patterned UV illumination through photomasks (Figure 3c). The characters “PBT” were first recorded on the film with high contrast by 254 nm UV irradiation. After they were completely erased by >510 nm visible light, the second group of characters “AIE”were written on the film. The fluorescent image was continuously exposed to 365 nm UV for 5 min without notable decrease in fluorescence contrast, indicating that fluorescence readout of **BTE-2PBT** in PMMA film could be achieved in a non-destructive manner.

## 3. Materials and Methods

### 3.1. General Methods

#### 3.1.1. Synthesis of **BTE-2PBT** Triad

All chemical reagents and solvents were obtained from a commercial supplier (Leyan-Shanghai Haohong Scientific Co. Ltd., Shanghai, China). All reactions were performed in AR grade solvents and monitored by thin layer chromatography on plates coated with 0.25 mm silica gel 60 F_254_ (Qingdao Haiyang Chemicals, Qingdao, China). TLC plates were visualized by UV irradiation (254 nm and 365 nm). Flash column chromatography employed silica gel (particle size 32–63 μm, Qingdao Haiyang Chemicals, Qingdao, China). NMR spectra were obtained with a Bruker AV-400 instrument (Bruker BioSpin, Faellanden, Switzerland) with chemical shifts reported in parts per million (ppm, *δ*) and referenced to CDCl_3_ or DMSO-*d*_6_. IR spectra were recorded on a Bruker Vertex-70 spectrometer. High-resolution mass spectra were obtained with a Bruker Dalton micrOTOF-Q II spectrometer (Bruker Optics, Billerica, MA, USA), and reported as *m*/*z*. Melting points were determined with a Thomas-Hoover melting point apparatus and uncorrected (Thomas Scientific, Swedesboro, NJ, USA).

#### 3.1.2. UV-Vis and Fluorescent Spectrometry

Absorption spectra of solution samples of **BTE-2PBT** (2 × 10^−5^ M) was recorded on an Aglient 8453 spectrophotometer (Agilent Technologies, Santa Clara, CA, USA). Absorption spectra of solid samples were recorded on a PerkinElmer Lambda 750 spectrophotometer (PerkinElmer, Waltham, MA, USA). Fluorescence spectra of **BTE-2PBT** solution (5 × 10^−^^5^ M) and solid samples were recorded on a Hitachi F-4600 fluorescence spectrophotometer (Hitachi, Yokohama, Kanagawa, Japan). Optical length of the quartz cell was 10 mm. Fluorescence quantum yields of **BTE-2PBT** solution (5 × 10^−5^ M) and solid samples were determined on a Hamamatsu absolute PL quantum yield spectrometer C11347 (Hamamatsu Photonics, Hamamatsu City, Shizuoka Pref, Japan). Fluorescence lifetimes were recorded on a Hamamatsu compact fluorescence lifetime spectrometer C11367 (Hamamatsu Photonics, Hamamatsu City, Shizuoka Pref, Japan).

#### 3.1.3. DLS Measurement

Particle size distribution was measured on a Nano Brook 90 Plus instrument (Brookhaven Instruments, Holtsville, NY, USA) equipped with a diode laser as a light source (λ = 632.8 nm). The scattered light from the sample solution was detected using a fixed angle (90°).

#### 3.1.4. Determination of Cyclization and Cycloreversion Quantum Yields

*Φ*_o-c_ and *Φ*_c-o_ values of **BTE-2PBT** in hexane [2 × 10^−5^ M] were measured according to a previously reported method with bis(2-methyl-1-benzothiophen-3-yl)hexafluorocyclopentene as the reference compound. A WFH-203B black-box type UV analyzer (Chitang Electronics, Shanghai, China) and an MVL-210M-visible light source (Mejiro Genossen, Akita, Japan) were employed for photo-induced cyclization and cycloreversion, respectively.

#### 3.1.5. Determination of **BTE-2PBT**’s PR at PSS

HPLC traces of **BTE-2PBT-*o*** and **BTE-2PBT-*c*** were recorded on an Agilent 1220 instrument equipped with a ZORBAX SIL analytical column (4.6 × 250 mm, 5 μm; Agilent Technologies) (flow rate = 1.0 mL/min; 3.0% isopropanol in CH_2_Cl_2_ over 15 min; UV detection at 320 nm (isosbestic point)).

#### 3.1.6. Photoerasing, Rewriting, and Nondestructive Reading of Fluorescent Images on **BTE-2PBT** PMMA Film

The PMMA film (M_W_ = ca. 110,000) containing **BTE-2PBT** (5 wt %) was prepared by spin coating on a 1 cm × 2 cm quatz glass slide. During the writing process, the film was irradiated through a photomask under a 254 nmWFH-204B UV lamp (Qiwei Instrument, Hangzhou, China) at a distance of 5 cm for 5 min. For erasing, the film was directly subjected to an MVL-210M-visible light source (Mejiro Genossen, Akita, Japan) at a distance of 5 cm for 3 min. Continuous non-destructive reading was performed under a 365 nm WFH-204B UV lamp (Qiwei Instrument, Hangzhou, China) at a distance of 5 cm for 5 min.

### 3.2. Synthesis and Characterization of PBT Compound (**1**)

*(4-(3-(Ethoxycarbonyl)-2-methyl-4H-benzo[4,5]thiazolo[3,2-a]pyrimidin-4-yl)phenyl)boronic acid* (**1**)**.** To a mixture of (4-formylphenyl)boronic acid (180 mg, 1.2 mmol), ethyl acetoacetate (195 mg, 1.5 mmol), 2-aminobenzothiazole (150 mg, 1.0 mmol) was added Hf(OTf)_4_ (8 mg, 0.01 mmol). The reaction was stirred in a sealed tube at 80 °C for 6 h. Flash column chromatography (DCM/MeOH = 20:1, *v*/*v*) afforded **1** as a yellow solid (327 mg, 83%); mp 241–242 °C. ^1^H-NMR (400 MHz, DMSO-*d*_6_): *δ* 8.00 (s, 2H), 7.74 (d, *J* = 7.8 Hz, 1 H), 7.66 (d, *J* = 7.8 Hz, 2 H), 7.42‒7.36 (m, 3H), 7.29 (dd, *J*_1_ = *J*_2_ = 7.5 Hz, 1H), 7.17 (dd, *J*_1_ = *J*_2_ = 7.5 Hz, 1H), 6.45 (s, 1H), 4.10‒4.00 (m, 2H), 3.34 (s, 3H), 1.20 (t, *J* = 7.1 Hz, 3H) ppm; ^13^C-NMR (100 MHz, DMSO-*d*_6_): *δ* 165.4, 162.6, 153.9, 142.9, 137.4, 134.1 (×2), 126.7, 125.9 (×2), 123.9, 122.7 (×2), 112.2, 102.6, 59.4, 56.6, 23.1, 14.0 ppm; IR (KBr): *v*_max_ 3346, 2975, 1714, 1608, 1519, 1470, 1427, 1414, 1368, 1342, 1323, 1272, 1241, 1212, 1165, 1085, 1046, 1018, 743 cm^−1^; HRMS (ESI+): *m/z* calcd for C_20_H_20_BN_2_O_4_S [M + H]^+^ 395.1231; found 395.1230.

### 3.3. Synthesis and Characterization of **BTE-2PBT**

*Diethyl 4,4′-(((perfluorocyclopent-1-ene-1,2-diyl)bis(5-methylthiophene-4,2-diyl))bis(4,1-phenyl-ene))bis(2-methyl-4H-benzo[4,5]thiazolo[3,2-a]pyrimidine-3-carboxylate)* (**BTE-2PBT**). To a solution of **1** (150 mg, 0.38 mmol) and dibromobisthienylethene **2** (80 mg, 0.15mmol) in THF/H_2_O (9:1, *v*/*v*, 5 mL) was added tetrakis(triphenylphosphine)palladium (11 mg, 0.009mmol) and Na_2_CO_3_ (97 mg, 0.92 mmol). The reaction was stirred at 80 °C for 12 h under nitrogen atmosphere. After the reaction was cooled to ambient temperature, THF was removed under reduced pressure. The residual aqueous solution was extracted with CH_2_Cl_2_ (20 mL) and washed with deionized H_2_O (10 mL × 2). The organic phase was combined, dried over Na_2_SO_4_, and concentrated in vacuo. Flash column chromatography on silica gel (CH_2_Cl_2_/MeOH = 30:1, *v*/*v*)afforded **BTE-2PBT** as a light greenish powder (108 mg, 67%); mp > 300 °C. ^1^H-NMR (400 MHz, CDCl_3_): *δ* 7.49‒7.39 (m, 10H), 7.26‒7.21 (m, 2H), 7.19 (s, 2H), 7.17‒7.07 (m, 4H), 6.41 (s, 2H), 4.25‒4.13 (m, 4H), 2.45 (s, 6H), 1.86 (s, 6H), 1.30 (t, *J* = 7.1 Hz, 6H) ppm; ^13^C-NMR (100 MHz, CDCl_3_): *δ* 166.9 (×2), 163.5 (×2), 155.2 (×2), 141.7 (×3), 141.2 (×2), 138.2 (×2), 136.2, 133.5 (×2), 132.3 (×2), 128.7 (×2), 128.0 (×4), 126.8 (×2), 126.0 (×5), 124.2 (×2), 124.0 (×2), 122.8 (×2), 122.4 (×2), 111.8 (×2), 103.0 (×2), 60.2 (×2), 57.5 (×2), 29.8 (×2), 23.9 (×2), 14.6 (×2), 14.5 (×2) ppm; ^19^F NMR (470 MHz, CDCl_3_): *δ* −110.03 (s, 4F), −131.85 (s, 2F); IR (KBr): *v*_max_ 3442, 1700, 1674, 1597, 1508, 1371, 1331, 1272, 1241, 1199, 1113, 1093, 1076, 988, 742 cm^−1^; HRMS (ESI+): *m/z* calcd for C_55_H_43_F_6_N_4_O_4_S_4_ [M + H]^+^ 1065.2066; found 1065.2063.

## 4. Conclusions

In summary, a photo-switchable AIE-active **BTE-2PBT** triad has been designed and synthesized. The triad well combines the photochromic properties of BTE moiety and AIE properties of PBT moieties. More importantly, the solid-state fluorescence of **BTE-2PBT** could be reversibly switched by alternate UV and visible light irradiation with ultra-high contrast. Repeated writing and erasing of fluorescent images on **BTE-2PBT** PMMA film with non-destructive readout indicate that this novel triad has the potential to be applied as a practical solid-state luminescent material.

## Figures and Tables

**Table 1:** Photochromic Parameters of **BTE-2PBT** and a Reference Bisthienylethene (**3**) ^a^.

Compound	λ_ab,o_ (*ε* = M^−1^·cm^−1^)	λ_ab,c_ (*ε* = M^−1^·cm^−1^)	*Φ*_o-c_ (%)	*Φ*_c-o_ (%)	PR (%)
**BTE-2PBT**	305 (3.91 × 10^4^)	584 (1.47 × 10^4^)	9.4	0.6	73
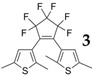	237 (2.15 × 10^4^)	503 (4.94 × 10^3^)	40	12	74

^a^ Determined in hexane (2.0 × 10^−5^ M) at 25 °C.

**Table 2:** Photophysical Data of **BTE-2PBT** and a Reference PBT AIEgen (**4**) ^a^.

Compound	State	λ_abs_ (nm) (*ε* = M^−^^1^cm^−^^1^)	λ_em_ (nm) (Δν)	*Φ*_F_ (%)	α_AIE_ (*Φ*_F,solid_/*Φ*_F,soln_)	τ (ns)
**BTE-2PBT**	soln	388 (2.91 × 10^4^)	477 (5560)	0.1	--	<0.1
	solid ^b^	413	475(1219)	1.2	12	0.78
	film	385	477(3307)	2.6	26	1.62
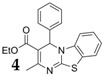	soln	384 (1.26 × 10^4^)	474 (3903)	0.5	--	<0.1
	solid ^c^	422	507 (2351)	39.1	78.2	5.75
	film	385	471 (4410)	4.2	8.4	2.05

^a^ Abbreviations: soln = THF solution (5 × 10^−5^ M); ε = molar absorptivity (M^−1^cm^−1^); Δ*ν* = Stokes shift (cm^−1^); *Φ*_F_ = fluorescence quantum yield determined using a calibrated integrating sphere; α_AIE_ = value of AIE effect; τ = fluorescence lifetime measured at room temperature in air; ^b^ Determined on amorphous sample; ^c^ Determined on crystalline sample.

## Data Availability

The data presented in this study are available in Supplementary Materials.

## Supplementary Materials

The following are available online, Figures S1 and S2: UV-Vis and fluorescence spectra of **BTE-2PBT**; Figure S3: HPLC traces of **BTE-2PBT-*o*** and **BTE-2PBT-*c***; Figures S4–S8: NMR spectra of **1** and **BTE-2PBT**; Figure S9: HRMS of **BTE-2PBT**.

## Sample Availability

Samples of the compounds **1** and **BTE-2PBT** are available from the authors.
