# Supplementary material for: Photo-Switchable Aggregation-Induced Emission of Bisthienylethene-Dipyrimido[2,1-b][1,3]benzothiazole Triad
Source: Molecules. 2021 Sep 4;26(17):5382. doi: 10.3390/molecules26175382 (PMC8433884; doi:10.3390/molecules26175382)
Supplement: Supplementary file 1 [file molecules-26-05382-s001.zip › molecules-1356068-supplementary.pdf]

Supporting Information

Photo-Switchable Aggregation-Induced Emission of Bisthienylethene-Dipyrimido[2,1-*b*][1,3]benzothiazole Triad

Shan-Shan Gong[1,2], Chun-Hong Zheng[2], Zhen-Zhen Chen[2], Dong-Zhao Yang[2], Mei Chi[2], Shou-Zhi Pu[1,2,3]*, and Qi Sun[2]*

[1]*College of Chemistry, Nanchang University, Nanchang, Jiangxi 330031, PR China*

[2]*Jiangxi Key Laboratory of Organic Chemistry, Jiangxi Science and Technology Normal University, Nanchang, Jiangxi 330013, PR China*

[3]*Department of Ecology and Environment, Yuzhang Normal University, Nanchang, Jiangxi 330103, PR China*

E-mails: pushouzhi@tsinghua.org.cn; sunqi@jxstnu.edu.cn

Table of contents

Figure S1. UV-Vis spectra of **BTE-2PBT** in solution (2×10^{-5} M), film, and solid state Page S2

Figure S2. Fluorescence spectra of **BTE-2PBT** in solution (5×10^{-5} M), film, and solid state Page S2

Figure S3. HPLC traces of **BTE-2PBT-*o*** and **BTE-2PBT-*c*** for determination of PR at PSS Page S2

Figure S4. ^{1}H NMR spectrum of **1** Page S3

Figure S5. ^{13}C NMR spectrum of **1** Page S3

Figure S6. ^{1}H NMR spectrum of **BTE-2PBT** Page S4

Figure S7. ^{13}C NMR spectrum of **BTE-2PBT** Page S4

Figure S8. ^{19}F NMR spectrum of **BTE-2PBT** Page S5

Figure S9. HRMS spectrum of **BTE-2PBT** Page S5

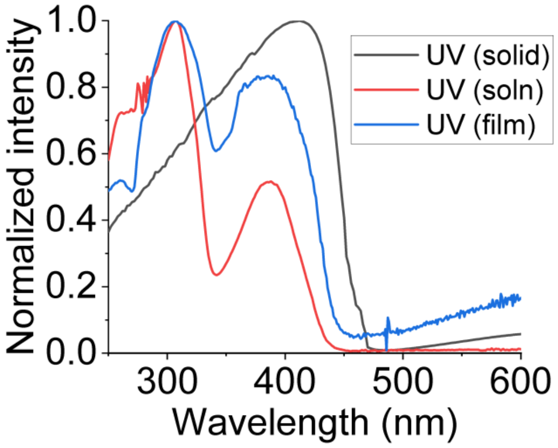

Figure S1. UV-Vis spectra of **BTE-2PBT** in solution (2×10^{-5} M), film, and solid state

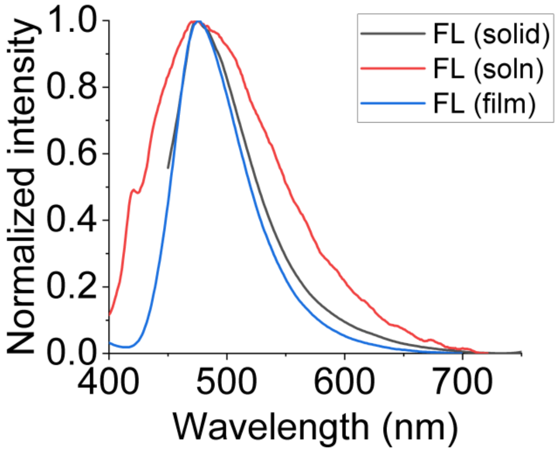

Figure S2. Fluorescence spectra of **BTE-2PBT** in solution (5×10^{-5} M), film, and solid state

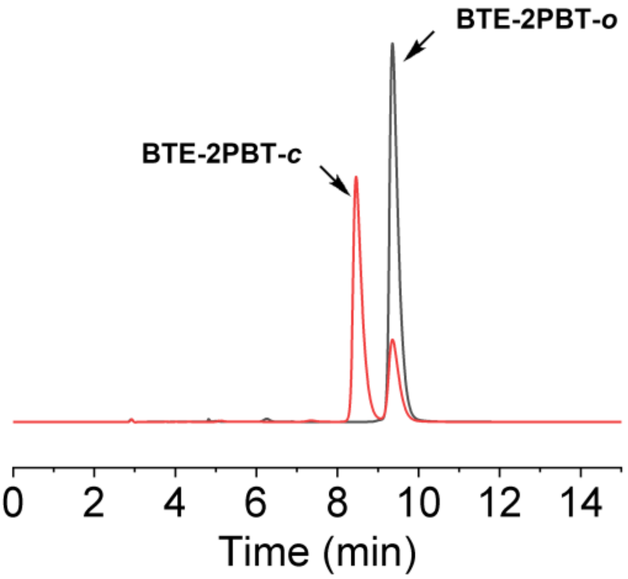

Figure S3. HPLC traces of **BTE-2PBT-*o*** and **BTE-2PBT-*c*** for determination of PR at PSS

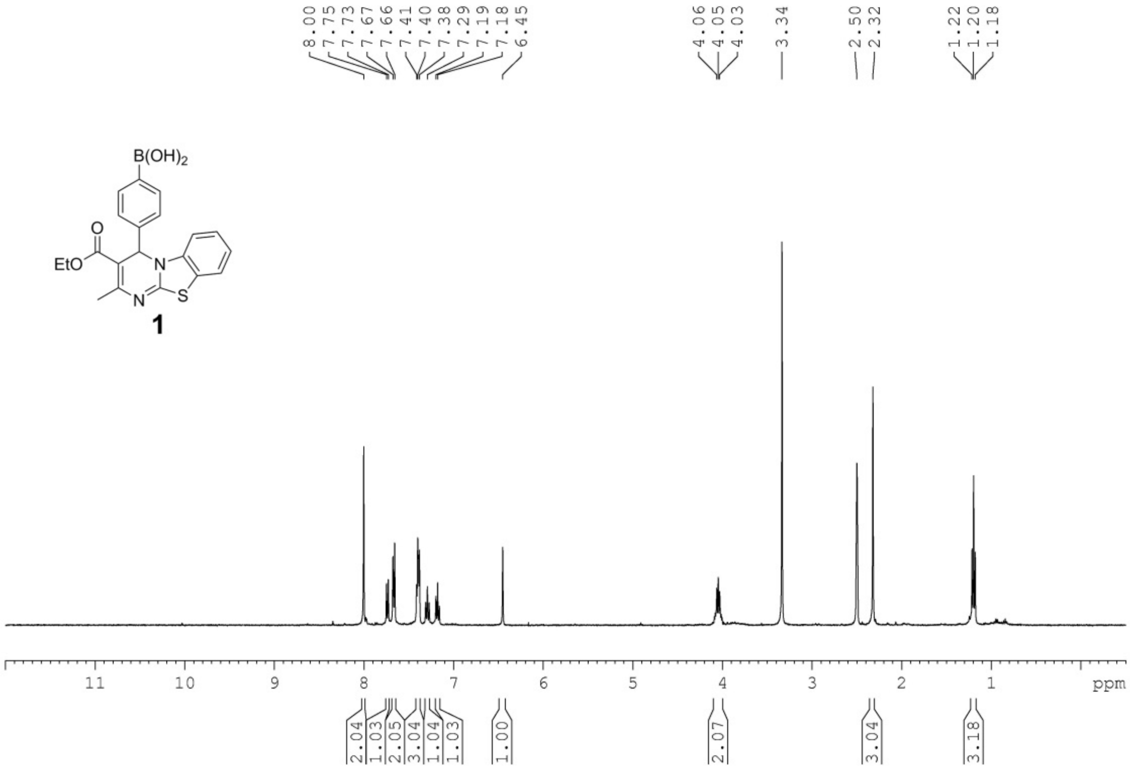

Figure S4. ^{1}H NMR spectrum of **1**

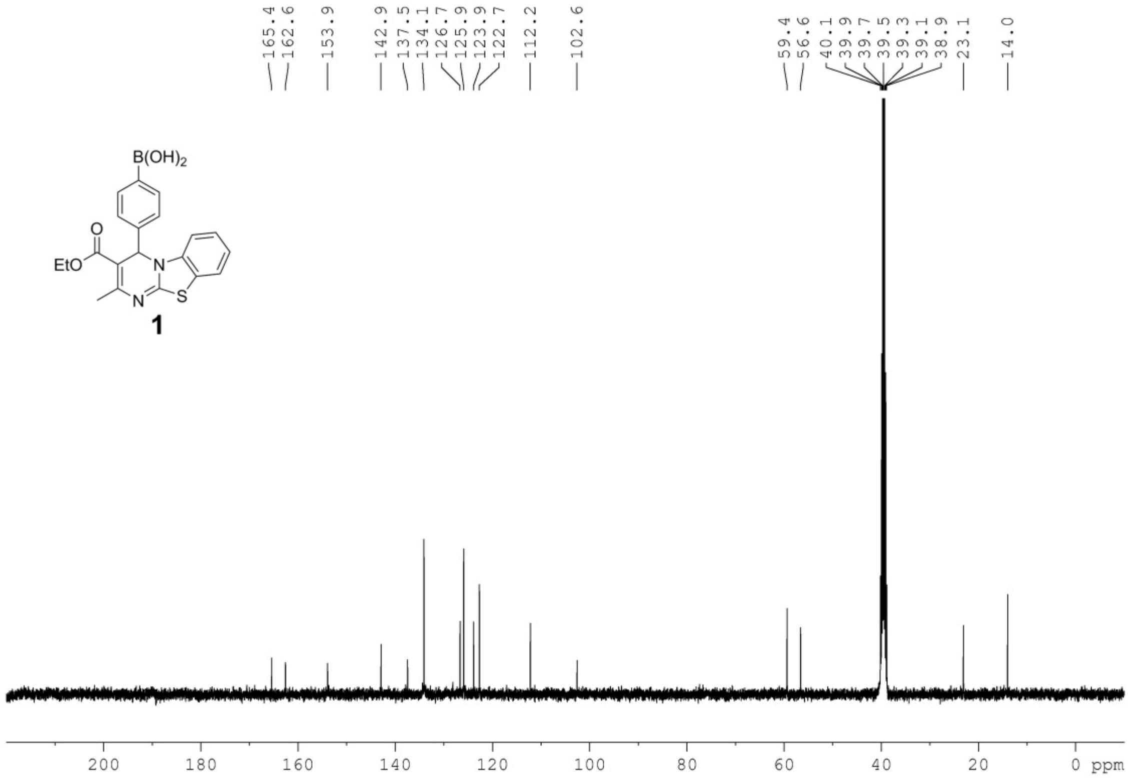

Figure S5. ^{13}C NMR spectrum of **1**

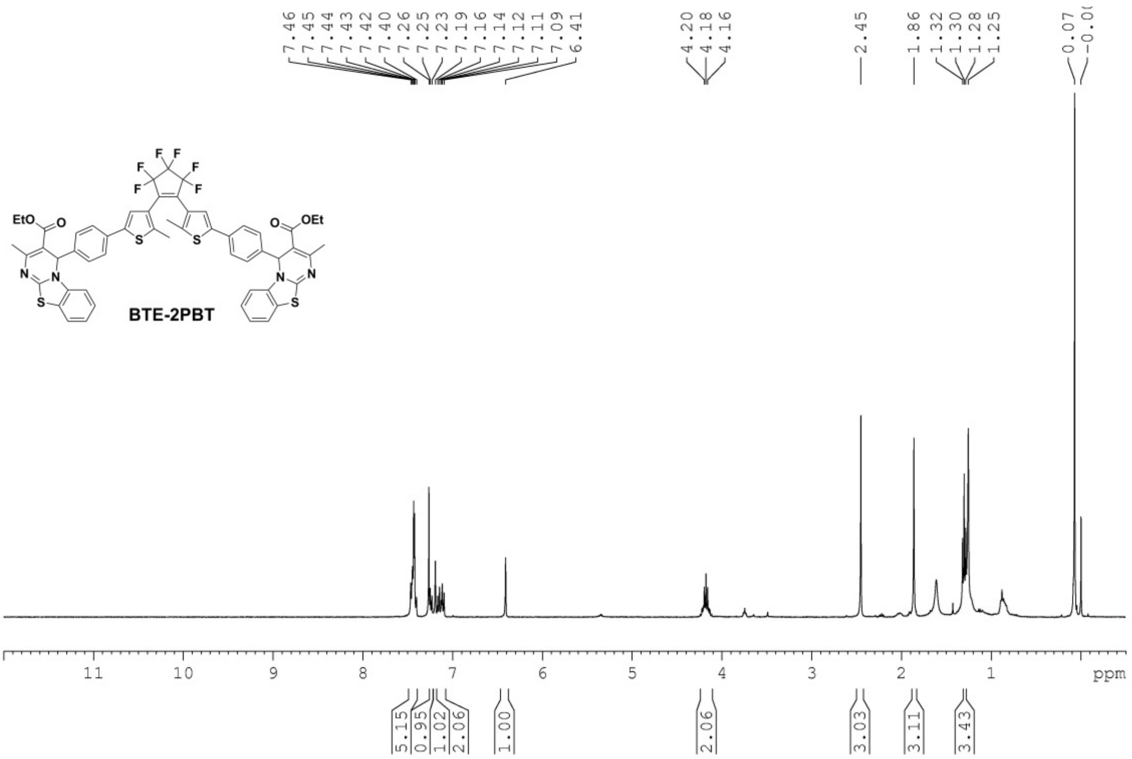

Figure S6. ^{1}H NMR spectrum of **BTE-2PBT**

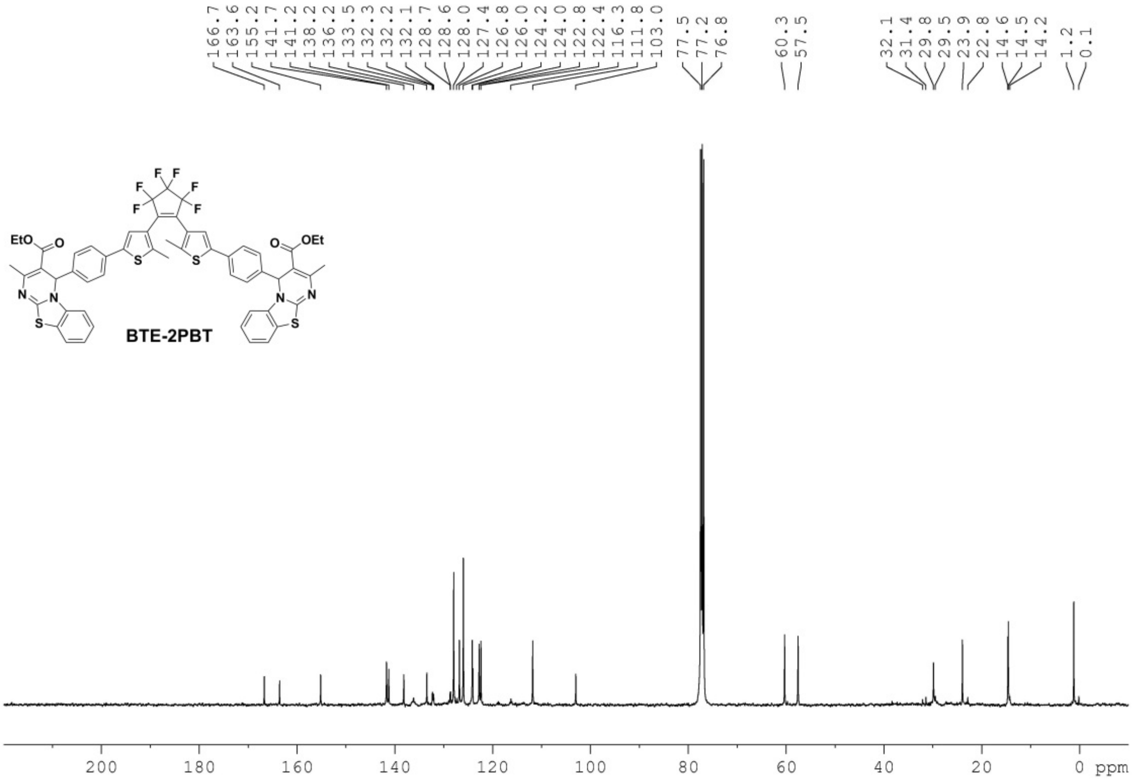

Figure S7. ^{13}C NMR spectrum of **BTE-2PBT**

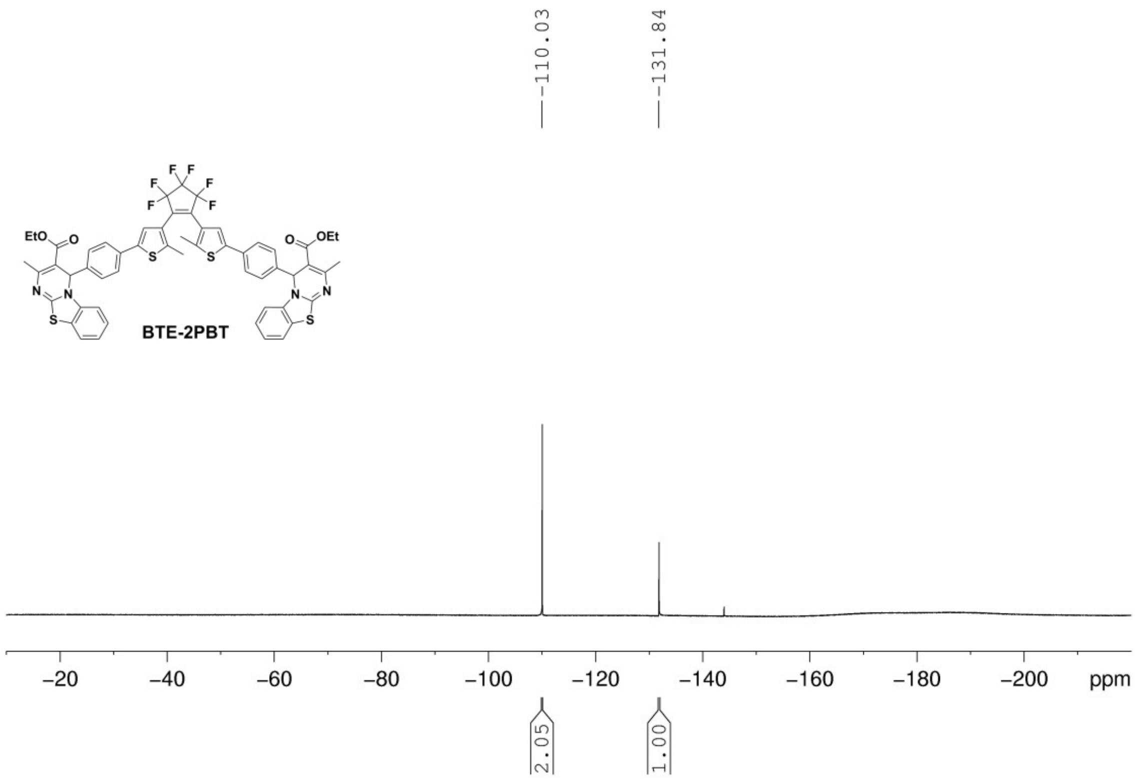

Figure S8. ^{19}F NMR spectrum of **BTE-2PBT**

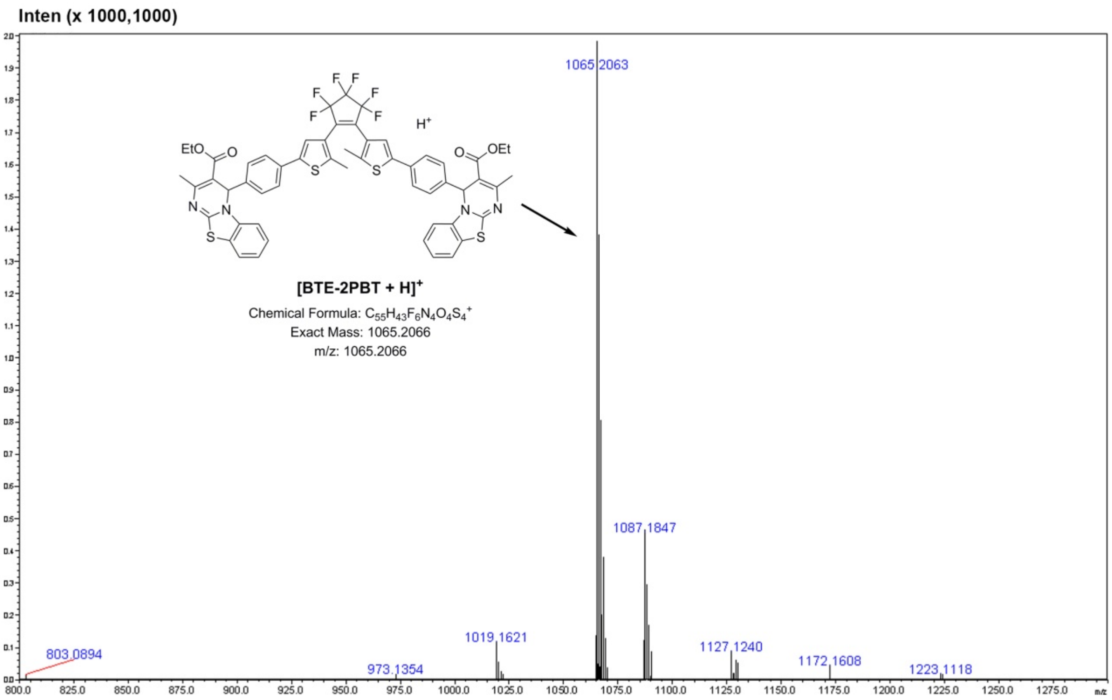

Figure S9. HRMS spectrum of **BTE-2PBT**
